# Beyond the gut: The overlooked impact of constipation on functional mobility and quality of life in community‐dwelling elders – a cross‐sectional study

**DOI:** 10.1111/ggi.70060

**Published:** 2025-05-08

**Authors:** Mucahit Oztop, Nesrin Yagcı

**Affiliations:** ^1^ Physiotherapy and Rehabilitation Department, Faculty of Health Science Burdur Mehmet Akif Ersoy University Burdur Turkiye; ^2^ School of Physical Therapy and Rehabilitation Pamukkale University Denizli Turkiye

**Keywords:** community‐dwelling elders, constipation, functional mobility, quality of life, rectal pain

## Abstract

**Aim:**

Constipation is common in the elderly and may impair functional mobility. This study examined its prevalence and impact on mobility in community‐dwelling elders.

**Methods:**

A total of 630 elders (340 females, 290 males; mean age 70.78 ± 4.88 years) with adequate cognitive function were evaluated. Constipation was defined via self‐report according to the Rome IV criteria and quantified with the Constipation Severity Instrument (CSI). Lower limb mobility was measured using the Five‐Times Sit‐to‐Stand Test (FTSST), while rectal pain and quality of life (QoL) were assessed using 10‐cm visual analog scales.

**Results:**

Constipation was self‐reported by 82.7% of participants. Compared with non‐constipated elders, those with constipation had significantly prolonged FTSST times, higher rectal pain scores, and lower QoL (all *P* < 0.001). Correlation analysis revealed that greater constipation severity (CSI scores) was strongly associated with poorer QoL (*r* = 0.71, *P* < 0.001) and rectal pain (*r* = 0.87, *P* < 0.001), and negatively correlated with cognitive function (*r* = −0.14, *P* < 0.001). Additionally, CSI scores were positively correlated with FTSST time (*r* = 0.21, *P* < 0.001) and rectal pain (*r* = 0.69, *P* < 0.001).

**Conclusions:**

Constipation in community‐dwelling elders is strongly linked to increased rectal pain, reduced lower limb functional mobility, and poorer QoL. These results support the need for integrated gastrointestinal and physiotherapy interventions, and future studies should use longitudinal and interventional designs to confirm causality and optimize treatment strategies. **Geriatr Gerontol Int 2025; 25: 799–805**.

## Graphical abstract

Constipation in community‐dwelling elders is associated with increased rectal pain, reduced lower limb functional mobility, and poorer quality of life. Our findings highlight the need for integrated gastrointestinal and physiotherapy interventions. Future research should employ longitudinal and interventional designs to confirm causality and optimize treatment strategies for aging populations.

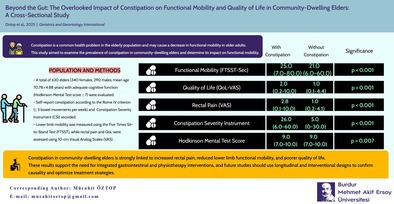

## Introduction

Constipation is a common condition affecting quality of life (QoL) at all ages. Functional constipation, as per the Rome IV criteria, involves fewer than three weekly bowel movements and symptoms such as straining or hard stools.^1^ Studies show that the mean prevalence of constipation is approximately 14%, ranging from 2% to 35%.^2^ According to Barberio *et al*., functional constipation prevalence is 10.1% in studies using the Rome IV criteria.^3^ A recent study conducted in the Turkish population found that 16.6% of adults were diagnosed with constipation and it identified being female, older age, and obesity as risk factors.^4^

Functional constipation may arise from diverse and complex pathophysiological mechanisms, such as colonic motility disorders, brain–gut axis dysfunction, and weak or altered sensorimotor pelvic floor muscle function.^5^, ^6^ Additionally, neurogenic dysregulation involving the central, autonomic, and enteric nervous systems contributes to functional constipation.^7^

Constipation is a major issue among individuals over 65, particularly in institutionalized elderly. A cross‐sectional study in northern Sweden found a 67% prevalence of constipation in this population.^8^ In the United States, 2.8 million visits to health care providers are made annually owing to constipation, and the annual direct cost for the management of constipation ranges from $1900 to $12 000 per patient.^9^

Various factors contribute to the increased incidence of constipation with aging. Changes in body systems, particularly the enteric nervous system, along with reduced mobility are among the key factors.^10^ Emmanuel *et al*., in a consensus statement, recommended that increasing mobility and exercise can relieve constipation symptoms.^11^ Given this, we sought to investigate whether constipation directly impacts functional mobility in elders living independently.

The aim of this study was to examine the constipation status in the elderly living at home and to determine the effect of constipation on functional mobility. We hypothesized that elders with constipation would have significantly lower functional mobility, higher rectal pain, and reduced QoL compared with non‐constipated individuals.

## Methods

### Participants

A total of 630 elderly participants (340 females; 290 males; age 65 years and over, mean age 70.78 ± 4.88 years) who scored 7 or above on the Hodkinson Abbreviated Mental Test were enrolled. A score of ≥7 indicates adequate cognitive function for reliable self‐reporting. Interviews were conducted with participants in their homes.

The inclusion criteria were: (i) over 65 years old and living in their own home; (ii) no communication or cognitive impairments that could affect assessment; (iii) good cognitive level (scoring 7 or above on the Hodkinson Abbreviated Mental Test). The exclusion criteria were: (i) neurological disorders causing constipation (e.g., Parkinson's, MS, spinal cord lesion, dementia); (ii) individuals who were bed‐bound (paralysis, immobility); (iii) illiteracy; (iv) use of medications such as anticholinergics or anti‐Parkinson drugs.

**Figure 1:**
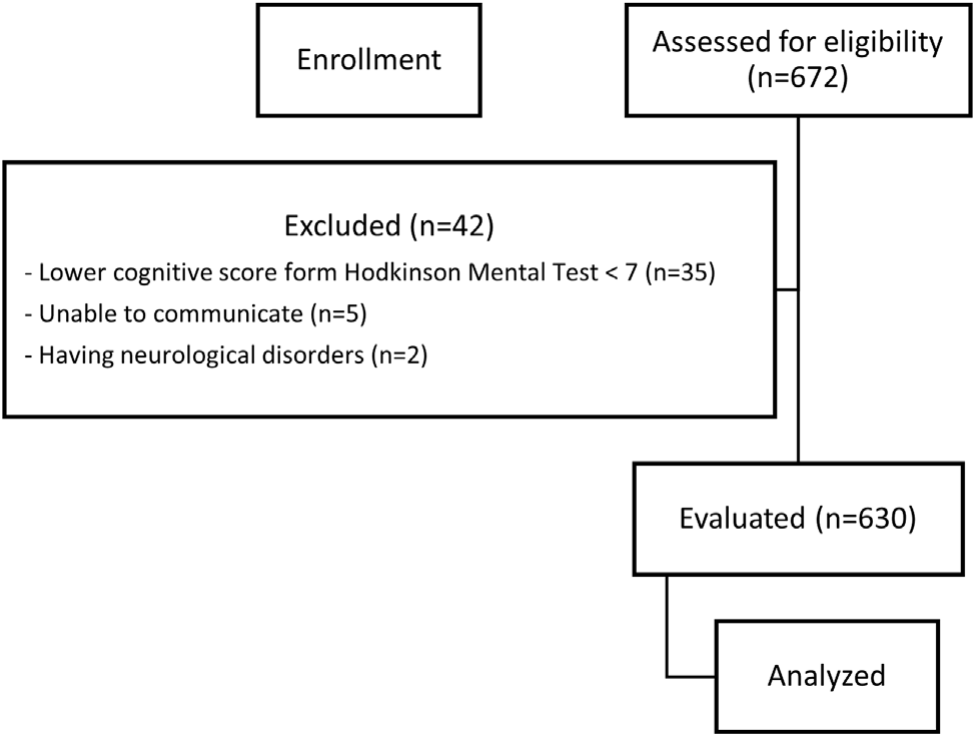
Flow chart of the enrollment of the study.

A flow chart of the enrollment of the study is given in Figure 1.

### Study design

This was a cross‐sectional study conducted via face‐to‐face interviews from September 2020 to February 2021. Sociodemographic data included age, sex, body mass index (BMI), marital status, education, smoking habits, toilet type, straining, and hemorrhoids. Constipation was self‐reported based on the Rome IV criteria (three or fewer bowel movements per week). The Constipation Severity Instrument (CSI) assessed severity, while lower limb mobility was evaluated with the Five‐Times Sit‐to‐Stand Test (FTSST). Rectal pain and overall QoL were measured with a 10‐cm visual analog scale (VAS).

This study was approved by Noninvasive Clinical Research Ethical Committee of Pamukkale University Denizli, Turkiye (60116787‐020/66564). The study was conducted in full agreement with national and international regulations and the Declaration of Helsinki. All participants provided informed verbal consent.

### Outcome measurements

#### Constipation Severity Instrument

The CSI was used to evaluate the severity of the constipation. The CSI was designed to evaluate defecation frequency and consistency as well as the level of straining experienced by individuals during bowel movements.^12^ There are three subscales of CSI: obstructive defecation, colonic inertia, and pain. Higher scores of CSI indicate more severe constipation. Kaya and Turan^13^ showed that the Turkish version of CSI is reliable and valid in determining constipation and grading its severity. The instrument provides a score between 0 (no problem) to 73 (severe constipation), and no cut‐off score was reported. Also, CSI includes three of the six Rome IV criteria: straining, incomplete evacuation, and manual maneuvers.^14^

#### Five‐Times Sit‐to‐Stand Test

The FTSST was used to assess functional mobility of the lower limbs. Participants started seated in an armless chair with a 43‐cm seat height, crossing their arms and leaning against the chair's back. The researcher demonstrated the test, including a full stand with extended hips and knees. Timing began when the participant said “go” and stopped when their hips returned to the seat after the fifth stand. Participants were instructed to stand and sit five times “as quickly as possible” without assistance. If a participant stopped and refused to continue, their score was excluded, with the reason for early quitting recorded.^15^ According to Albalwi *et al*., the cut‐off score for the FTSST is 11.5 s for the age group of 65–74 years.^16^ According to de Melo *et al*., inter‐rater (Intraclass Correlation Coefficient [ICC] 0.92, 95% confidence interval [CI] 0.89–0.94) and intra‐rater (ICC 0.95, 95% CI 0.93–0.96) reliability were found to be excellent, and higher scores were associated with lower muscle strength.^17^

#### Visual analog scale

A 10‐cm VAS was used to assess rectal pain severity during stool production. Participants marked the severity of pain on the scale, with 0 indicating no pain and 10 representing unbearable pain. The marked point was then measured with a ruler and recorded.^18^

A VAS was also used to assess QoL changes due to constipation. Participants marked a point on a 10‐cm horizontal line, with 0 indicating no impact on QoL and 10 indicating a significant impact. The marked point was then measured in centimeters and recorded. According to de Boer *et al*., VAS demonstrates good validity and excellent reliability for assessing global QoL.^19^ A single‐item VAS was selected for its simplicity and validated use in assessing global QoL.

### Statistical analysis

All statistical analyses were performed using Statistical Package for the Social Sciences (SPSS) for Windows, version 25.0 (IBM Corp., Armonk, NY). Continuous variables were expressed as mean ± standard deviation (SD), and categorical variables as numbers and percentages. The Shapiro–Wilk test was used to verify the normality of the main variables. The Mann–Whitney *U* test was used to compare numerical variables in paired groups because our data did not conform to a normal distribution. Spearman's rank correlation analysis was performed to assess the relationships among variables, considering the non‐parametric nature of the data. Correlation coefficients (Spearman's rho) were calculated for QoL, CSI total score, FTSST time, rectal pain (VAS), and Hodkinson Mental Test score. Significance was accepted as *P* < 0.05.

## Results

Figure 1 illustrates the flow chart of patients who met the inclusion/exclusion criteria. The mean age of participants was 70.78 ± 4.88 years. Of the participants, 54% were male, 84.8% were married, 84.4% had 8 years or less of education, and 59.4% had never smoked. The majority (62.2%) preferred a seated toilet, and 82.7% reported experiencing constipation (Table 1).

**Table 1:** Demographics and constipation variables

Characteristics	Mean ± SD
Age (years)	70.78 ± 4.88
BMI (kg/m^2^)	27.49 ± 4.50

	*n* (%)
Sex	
Female	290 (46)
Male	340 (54)
Marital status	
Single	96 (15.2)
Married	534 (84.8)
Education level	
≤8 years	532 (84.4)
>8 years	98 (15.6)
Smoking habit	
Smoking actively	132 (21)
Smoked and stopped	124 (19.7)
Never used	374 (59.4)
Toilet type	
Squat toilet	213 (33.8)
Seated toilet	417 (66.2)
Constipation	
Yes	521 (82.7)
No	109 (17.3)

BMI, body mass index; SD, standard deviation.

Table 2 shows the distribution of defecation history and BMI of participants by sex. The majority of both males (67.7%) and females (67.1%) with constipation reported experiencing difficulty passing stools (straining). Only 16% of participants with constipation (female: 17.4%; male: 14.3%) reported manually assisting stool output. Additionally, 20.3% of participants with constipation had hemorrhoids. The BMI values of most female (43.8%) and male (45.9%) participants with constipation were categorized as overweight. (Table 2).

**Table 2:** Distribution of defecation history and body mass index in the elderly with constipation by sex

Variables	Constipation (*n* = 488)		*n*	%
Difficulty passing stool	Yes (*n* = 313)	Female	162	61.1
		Male	151	67.7
	No (*n* = 175)	Female	103	38.9
		Male	72	32.3
Manual removal of stool	Yes (*n* = 78)	Female	46	17.4
		Male	32	14.3
	No (*n* = 410)	Female	219	82.6
		Male	191	85.7
Hemorrhoids	Yes (*n* = 99)	Female	48	18.1
		Male	51	22.9
	No (*n* = 389)	Female	217	81.9
		Male	172	77.1
BMI (kg/m^2^)	Female (*n* = 265)	Underweight <18.5	‐	‐
		Normal 18.5–24.9	80	27.6
		Overweight 25.0–29.9	127	43.8
		Obese ≥30	83	28.6
	Male (*n* = 223)	Underweight <18.5	‐	‐
		Normal 18.5–24.9	81	35.4
		Overweight 25.0–29.9	105	45.9
		Obese ≥30	43	18.8

BMI, body mass index.

**Figure 2:**
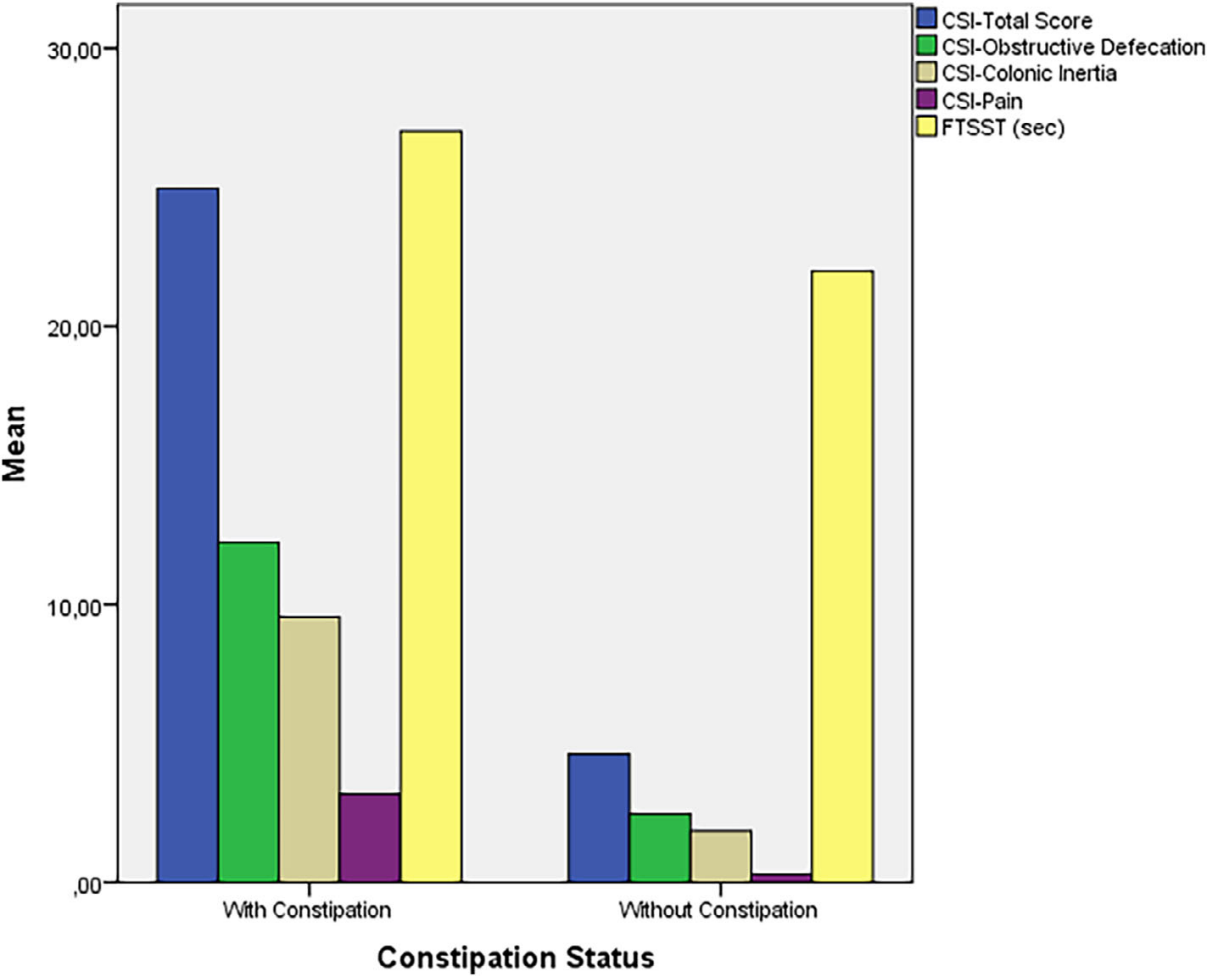
Distribution of Constipation Severity Instrument (CSI) subscale scores and Five‐Times Sit‐to‐Stand Test (FTSST) scores according to constipation status.

Table 3 presents a comparison of rectal pain, QoL impact, and CSI scores based on constipation status. Elderly participants with constipation experienced significantly higher rectal pain severity and QoL impact than did those without constipation (*P* < 0.001). The CSI total scores were also higher in those with constipation, with a highly significant difference (*P* < 0.001). This reflects a substantial increase in symptom burden. When examining the CSI subscores, those with constipation scored higher in the obstructive defecation, colonic inertia, and pain subgroups, and these differences were highly significant (*P* < 0.001). The FTSST duration was longer in participants with constipation than in those without, with a statistically significant difference (*P* < 0.001) (Fig. 2). Additionally, participants with constipation had significantly lower scores on the Hodkinson Mental Test compared with those without constipation (Table 3).

**Table 3:** Comparison of rectal pain, effect on QoL and Constipation Severity Instrument scores of the participants according to their constipation status

	Total (*n* = 630)	With constipation (*n* = 521)	Without constipation (*n* = 109)	*w*	*z*	*P* *
Variables	Median (Min–Max)	Median (Min–Max)	Median (Min–Max)			
Rectal pain (VAS, cm)	2.5 (0.1–10.0)	2.8 (0.1–10.0)	1.0 (0.2–4.10)	3925.5	−5.736	0.0001
Effect on QoL (VAS, cm)	2.0 (0.1–9.90)	2.0 (0.2–10.0)	1.0 (0.1–4.4)	3173.0	−5.304	0.0001
CSI total score	25.0 (0–60.0)	26.0 (6.0–60.0)	5.0 (0–30.0)	7258.5	−15.705	0.0001
CSI subscale score						
Obstructive defecation	12.0 (0–28.0)	13.0 (0–28.0)	3.0 (0–19.0)	8573.0	−14.965	0.0001
Colonic inertia	10.0 (0–23.0)	11.0 (0–23.0)	1 (0–11.0)	10 335.5	−13.944	0.0001
Pain	3.0 (0–13.0)	3.0 (0–13.0)	0 (0–5.0)	16 150.5	−10.869	0.0001
FTSST (s)	25.0 (6.0–80.0)	25.0 (7.0–80.0)	21.0 (6.0–60.0)	26 637.5	−4.488	0.0001
Hodkinson mental test score	9.0 (7.0–10.0)	9.0 (7.0–10.0)	9.0 (7.0–10.0)	159 867.0	−2.716	0.007

CSI, Constipation Severity Instrument; FTSST, Five‐Times Sit‐to‐Stand Test; Max, maximum; Min, minimum; QoL, quality of life; VAS, visual analog scale.

* Mann–Whitney *U* test.

Spearman's rank correlation analysis revealed significant low to moderate relationships among QoL, CSI total score, FTSST time, rectal pain (VAS), and Hodkinson Mental Test score (Table 4). Constipation impact on QoL was negatively correlated with Hodkinson Mental Test score (*r* = −0.136, *P* < 0.001) and positively correlated with CSI total score (*r* = 0.713, *P* < 0.001), FTSST time (*r* = 0.227, *P* < 0.001), and rectal pain (*r* = 0.869, *P* < 0.001). Higher QoL VAS scores indicates significant negative impact of constipation on QoL. Positive correlation of CSI, rectal pain and QoL shows negative impact of constipation and rectal pain severity on quality of life. Similarly, CSI total score was positively associated with FTSST time (*r* = 0.208, *P* < 0.001) and rectal pain (*r* = 0.685, *P* < 0.001) but negatively correlated with Hodkinson Mental Test score (*r* = −0.164, *P* < 0.001). Moreover, FTSST time was positively correlated with rectal pain (*r* = 0.207, *P* < 0.001) and negatively correlated with Hodkinson Mental Test score (*r* = −0.165, *P* < 0.001). Rectal pain also exhibited a significant negative correlation with Hodkinson Mental Test score (*r* = −0.130, *P* < 0.001).

**Table 4:** Correlation of QoL, Constipation Severity Instrument scores, functional mobility scores, rectal pain, and Hodkinson Mental Test scores of the participants

Variable	QoL	CSI total score	FTSST score (s)	Rectal pain (VAS)	Hodkinson Mental Test score
QoL	‐	0.713**	0.227**	0.869**	−0.136**
CSI total score		‐	0.208**	0.685**	−0.164**
FTSST score (s)			‐	0.207**	−0.165**
Rectal pain (VAS)				‐	−0.130**
Hodkinson Mental test score					‐

CSI, Constipation Severity Instrument; FTSST, Five‐Times Sit‐to‐Stand Test; QoL, quality of life; VAS, visual analog scale. *P < 0.01; **P < 0.001 Spearman's rho.

These findings suggest that greater constipation severity, impaired functional mobility, and increased rectal pain are associated with lower cognitive function and reduced QoL in the study population.

## Discussion

In this study, which investigates constipation status in elderly individuals living at home and its relationship with functional mobility and QoL, our data indicate that the vast majority of elderly participants suffer from constipation. Most of the elderly included in this study reported experiencing difficulty passing stools, with some resorting to manual intervention for stool removal. Additionally, our findings show that rectal pain is a common complaint among both constipated and non‐constipated elderly individuals. Furthermore, lower limb functional mobility and QoL are significantly lower in elderly individuals with constipation than in those without.

Constipation is a common problem in the elderly, whether they live at home or are institutionalized, although different percentages are given in different studies.^2^, ^3^, ^8^, ^9^ In our study, the rate of constipation in the elderly living at home was higher than the rates stated in the literature. The high prevalence may reflect the self‐reporting of fewer than three stools per week, or factors such as low socioeconomic status, malnutrition, and low fiber intake.^20^ Also, a higher BMI score is a risk factor for constipation, and our data show that 72% of females and 65% of males are overweight or obese.

Straining and manual removal are common symptoms of constipation and were detected in the elderly who participated in our study. In a review examining the differences in constipation characteristics by sex, it was noted that straining and manual removal were significantly more common in women.^21^ As mentioned in the review above, it was observed in our study that women used the manual removal method more frequently, but, contrary to the literature, the rate of straining was found to be higher in men. This may reflect cultural toileting habits or sampling differences. Women are at higher risk of injury to the pelvic floor muscles and nerves involved in defecation, and as a result, may need to manually assist in bowel movements more frequently due to difficulties caused by pelvic surgery or childbirth.^22^ In a study by Werth *et al*., when self‐reported constipated and non‐constipated patients were compared, a significantly higher rate of hemorrhoids (22.0%–6.9%) was found in the constipated group.^3^ The results obtained in our study are in agreement with the study of Werth *et al*., and the incidence of hemorrhoids in the elderly with constipation is approximately 20%. Although the etiology of hemorrhoids and the relationship between hemorrhoids and constipation are not clearly known, the current belief is that constipation may cause chronic straining and hard stools, resulting in degeneration of the anal canal and distal displacement of anal cushions.^23^

Varma *et al*. found a significant difference between the groups in the CSI total and subscores in the group with and without constipation in their study, CSI total and subscores were significantly higher in the group with constipation compared to the group without constipation. They also found an inverse relationship between CSI total score and QoL and between the pain subscale and physical component of QoL. In our study, we evaluated rectal pain intensity and QoL with a VAS in addition to the CSI. We found a significant difference between groups with and without constipation in terms of rectal pain intensity and QoL. There is no study in the literature that has examined rectal pain and QoL in the population with constipation. Our data showed that rectal pain intensity and QoL were worse in the group with constipation, agreement with Varma *et al*.'s findings.^12^

Emmanuel *et al*., in their consensus statement on constipation, stated that decreased physical activity due to poor mobility and frailty is an important problem in relation to constipation in the elderly.^11^ In the same consensus statement, it was recommended that, in addition to fiber‐rich food consumption and sufficient fluid intake, mobility should be increased to a level conforming to age in non‐medication‐related constipation. Our findings demonstrate that lower limb mobility in constipated elders was significantly reduced compared with that in non‐constipated individuals, consistent with prior studies. As stated in the literature, low mobility is seen as an important risk factor for constipation and should be considered, especially in the fight against constipation in the elderly.^11^, ^24^

The present study's findings are in line with the existing literature on constipation in the elderly. Our observation of a strong positive correlation between constipation severity and rectal pain supports previous reports linking excessive straining and incomplete evacuation with increased anorectal discomfort.^25^, ^26^ Furthermore, the association between constipation severity and impaired functional mobility is consistent with studies showing that diminished lower limb strength due to sarcopenia, difficulty in walking, and balance deficits can both predispose to and result from chronic constipation.^27^, ^28^, ^29^ Notably, we found that lower cognitive function was negatively correlated with constipation severity, rectal pain, QoL, and functional mobility. Kang *et al*. reported that constipation could be a marker of cognitive impairment in Parkinson's disease, and Jank *et al*. stated that functional constipation is associated with a decline in word‐list recognition.^30^, ^31^ According to Wan *et al*., a decline in cognitive function might be due to changes in the microbiome.^32^ Finally, the inverse relationship between QoL and constipation severity echoes prior research demonstrating that constipation significantly diminishes daily functioning and overall well‐being.^33^, ^34^

Constipation could significantly impact functional mobility through pain, bloating, and fatigue, leading to reduced physical activity. Straining and increased intra‐abdominal pressure may contribute to pelvic floor dysfunction, affecting posture and gait. Additionally, abdominal discomfort can alter movement mechanics, increasing fall risk in elders.^5^

Constipation affects functional mobility through pain, inflammation, and brain–gut axis dysregulation, creating a vicious cycle. Managing constipation with hydration, diet, and activity may improve mobility. Future studies should include objective activity and dietary assessments to enhance understanding and rehabilitation strategies.^6^, ^7^

This study is limited by the lack of data on medication use, diet, fiber intake, hydration status, and physical activity, which may affect constipation rates. Its cross‐sectional design prevents causal inferences, and reliance on self‐reported data may introduce recall bias and subjectivity.

This study's strengths include a comprehensive assessment of constipation symptoms, rectal pain, and QoL using VAS, areas rarely explored in the literature. Validated tools enhance reliability, and the findings highlight the need for an integrated clinical approach. Future longitudinal studies with objective physical activity measures are recommended.

## Author contributions

All authors contributed to the study conception and design. Material preparation, data collection, and analysis were performed by NY and MO. The first draft of the manuscript was written by NY and MO, and all authors commented on present versions of the manuscript. All authors read and approved the final manuscript.

## Funding information

The authors did not receive support from any organization for the submitted work.

## Disclosure statement

The authors declare no conflicts of interest or financial support, confirm that the manuscript has not been submitted elsewhere, and state that all authors have approved it as basic research.

## Ethics statement

This study was approved by the Noninvasive Clinical Research Ethical Committee of Pamukkale University Denizli, Turkiye (60116787‐020/66564).

## Patient consent statement

All procedures performed in studies involving human participants were in accordance with the ethical standards of the institutional and/or national research committee and with the 1964 Helsinki Declaration and its later amendments or comparable ethical standards. Informed consent of all participants was given.

## Clinical trial registration

This observational study lacks interventions, experimental treatments, or randomization, so clinical trial registration was not required.

## Data Availability

The data that support the findings of this study are available on request from the corresponding author. The data are not publicly available due to privacy or ethical restrictions.
